# Applications of Machine Learning Models to Predict and Prevent Obesity: A Mini-Review

**DOI:** 10.3389/fnut.2022.933130

**Published:** 2022-07-05

**Authors:** Xiaobei Zhou, Lei Chen, Hui-Xin Liu

**Affiliations:** ^1^Health Sciences Institute, China Medical University, Shenyang, China; ^2^Liaoning Key Laboratory of Obesity and Glucose/Lipid Associated Metabolic Diseases, China Medical University, Shenyang, China; ^3^Institute of Life Sciences, China Medical University, Shenyang, China

**Keywords:** nutrition, obesity, machine learning, algorithm, genetics, environment

## Abstract

Research on obesity and related diseases has received attention from government policymakers; interventions targeting nutrient intake, dietary patterns, and physical activity are deployed globally. An urgent issue now is how can we improve the efficiency of obesity research or obesity interventions. Currently, machine learning (ML) methods have been widely applied in obesity-related studies to detect obesity disease biomarkers or discover intervention strategies to optimize weight loss results. In addition, an open source of these algorithms is necessary to check the reproducibility of the research results. Furthermore, appropriate applications of these algorithms could greatly improve the efficiency of similar studies by other researchers. Here, we proposed a mini-review of several open-source ML algorithms, platforms, or related databases that are of particular interest or can be applied in the field of obesity research. We focus our topic on nutrition, environment and social factor, genetics or genomics, and microbiome-adopting ML algorithms.

## Introduction

Obesity is considered to be a chronic progressive disease caused by a combination of multiple determinants, including biological, genetic, social, environmental, and behavioral factors (1, 2). Research on obesity and related diseases has received attention from government policymakers; interventions targeting nutrient intake, dietary patterns, and physical activity are deployed globally. Considerable current effort has been made by the computer community and industry to apply artificial intelligence (AI) technology in the field of biology and biomedicine (3, 4); a possible solution is to use modified machine learning (ML) algorithms to detect obesity disease biomarkers or discover intervention strategies to optimize the weight loss results. ML is seen as part of AI, allowing software applications to predict outcomes without being interpretable (5). ML models can be summarized as two categories: (I) supervised learning models relying on labeled data to train a function that can finish the prediction tasks, and (II) unsupervised learning models focusing on summarizing the characteristics of data, such as dimensionality reduction analysis.

Machine learning models have been successfully used in many studies on obesity to predict obesity rates and identify the risk factors in samples of interest (6–9). In the field of ML research, it is important and necessary to share the code used in research to check the reproducibility of a related work. In addition, open-source tools could greatly improve the efficiency of similar studies by other researchers. However, these studies ignored one significant thing: They did not share their ML methodologies and frameworks with the public for use by other researchers. At this stage, obesity-related research is considered a public health field and is mostly published in certain professional or medical journals, and these journals do not have strict requirements on the openness of the algorithms or frameworks used in the manuscripts, which are far less than the requirements for data sharing. After matching the keyword “github” (which is the world's most popular public code repository) in 491 abstract texts of obesity-ML-related research [using the PubMed search strategy “Obesity and Machine Learning and” (“1990/01/ 01” [PDAT]: “2022/04/01” [PDAT])], only one related paper was found. Open-source tools are tools for which the original source code is freely available and can be redistributed and modified (10). As a new and booming branch of computer science, ML inherits the tradition of open sharing in the computer community; relevant top journals or conferences have requirements for code sharing of published papers, and most researchers are also willing to upload their codes to the software source code repository.

**Figure 1:**
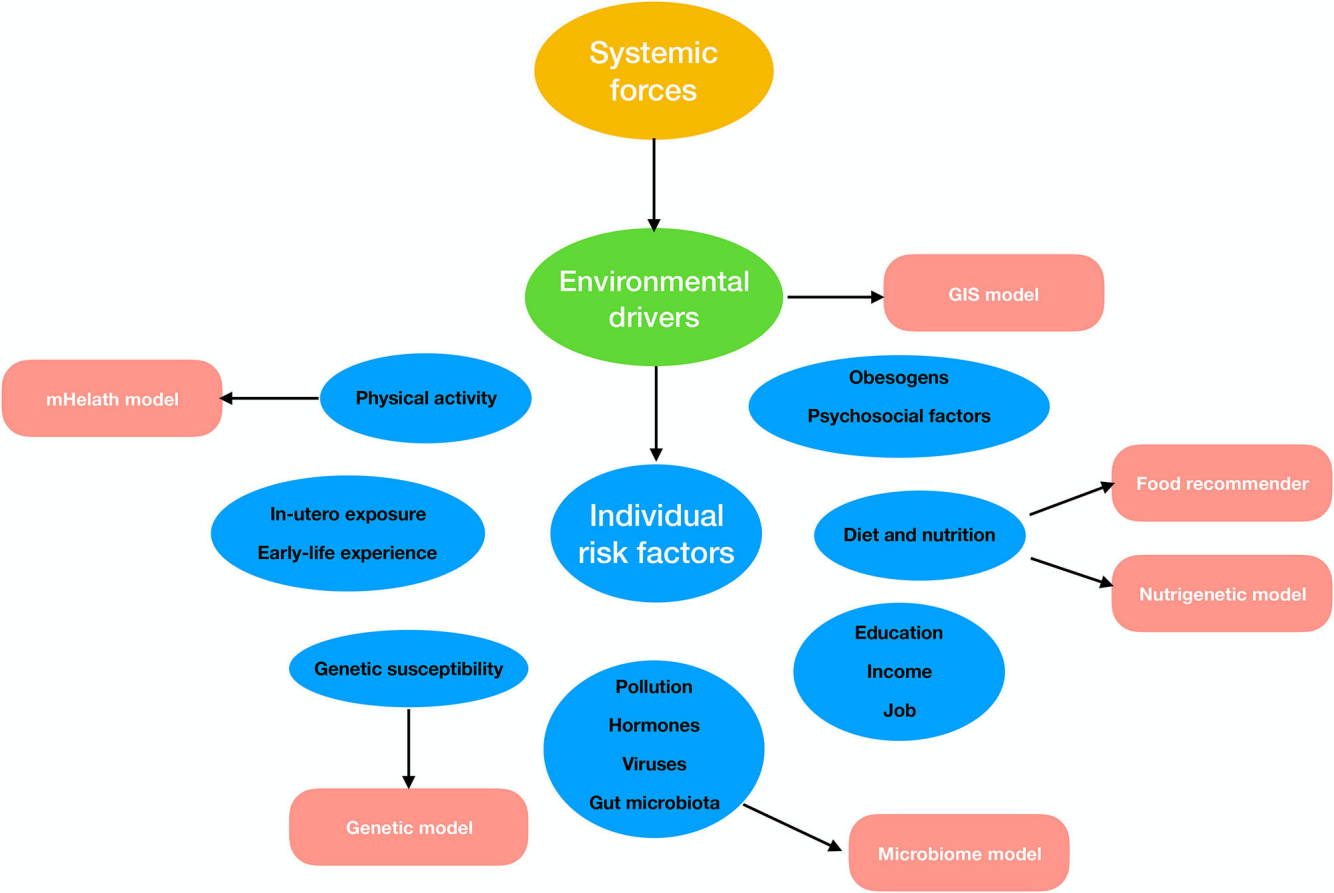
Possible machine learning (ML) applications according to risk factors leading to obesity.

Several existing reviews focused on the detailed illustration of the status (including the fundamentals, strength, limitation, and evaluation metric) of ML methods in obesity, while ignoring the discussion on the openness of algorithms (7, 11–13). To address this point, we proposed a mini-review for collecting open-source ML algorithms or platforms particularly focusing on the field of obesity research or that could be used in related problems in this article. Since the etiology and pathogenesis of obesity are extremely complex, we set the starting point of our study on the prior knowledge of the current authoritative study of obesity epidemiology (14). The traditional approach to epidemiological obesity research is to study numerous risk citations for obesity through specialized cohorts or epidemiological surveys (14). These customized ML tools for these specialized studies did not take our interest because of their low generality. We focused on ML algorithms related to obesity into several segments, including diet and nutrition, physical activity, geographic environment, genetics or genomics, and microbiome, in which the required data of ML models are available from the database (e.g., GEO database) or public platform (Google Map) without special input (Figure 1). In addition, we collected detailed information of 25 open-source ML algorithms or models applied in obesity or that could be used in related filed, including the project name, the relative website, applicable data types, and the simplified description of usage (Table 1).

**Table 1:** Detailed information on these ML algorithms.

**Name**	**Usage**	**Core algorithm**	**Input data**	**URL**
PFoodReq	Food recommendation system	Knowledge graph (KG) & question answering (QA) & recipe retrieval	Text	https://github.com/hugochan/PFoodReq
FlavorGraph	Food recommendation system	KG & recipe retrieval & compound food relationship	Vector	https://github.com/lamypark/FlavorGraph
DeepFood	Food recommendation system	Deep learning (DL)& image recognition & recipe retrieval	Image	https://github.com/deercoder/DeepFood
Market2Dish	Food recommendation system	DL& image recognition & recipe retrieval	Image	https://github.com/WenjieWWJ/FoodRec
LC-N2G	Nutrigenetics analysis method	Statistical methods	Gene expression data (GSE85998)	https://sydneybiox.github.io/LCN2G/
NutriGenomeDB	Nutrigenetics platform	None	None	https://github.com/rmartin84/NutriGenomeDB (locally deployment)
MapMetadataEnrichment	GIS satellite image analysis tool	DL	Satellite image	https://github.com/geoai-lab/MapMetadataEnrichment
GWmodel	An R package for exploring spatial heterogeneity	Spatial regression models	POI data	https://cran.r-project.org/web/packages/GWmodel/index.html
Schema	Digital intervention	None	None	https://github.com/schema-app/schema
mHealthDroid	mHealth platform	None	None	https://github.com/mHealthTechnologies/mHealthDroid
MobileCoach	Digital intervention	None	None	https://github.com/schlegel11/MobileCoach
PGS Catalog	Polygenic score (PRS) database	None	None	https://www.pgscatalog.org/
Impute.me	Platform for direct-to-consumer genetic testing	none	23AndMe	https://github.com/lassefolkersen/impute-me
DeepVariant	Deep learning-based variant caller	DL	BAM or CRAM	https://github.com/google/deepvariant
NeuralCVD	Cardiovascular risk predictor	Survival machine algorithm	UK Biobank data	https://github.com/thbuerg/NeuralCVD
DeepCOMBI	AI tool for analysis of GWAS data	DL	GWAS data	https://github.com/AlexandreRozier/DeepCombi
DeepMicro	Taxonomic classifier	DL	Microbe data (csv)	https://github.com/minoh0201/DeepMicro
DeepMicrobes	Taxonomic classifier	DL	Microbe data (fasta)	https://github.com/MicrobeLab/DeepMicrobes
SortMeRNA	Taxonomic classifier	ML	Microbe data (fasta)	https://github.com/biocore/sortmerna
q2-feature-classifier	Taxonomic classifier	ML	Microbe data (fasta)	https://github.com/qiime2/q2-feature-classifier
Swarm	Taxonomic classifier	ML	Microbe data (fasta)	https://github.com/torognes/swarm
GEDFN	Microbial biomarker' identification	DL	OTU & IBD	https://github.com/MicroAVA/GEDFN
MDeep	Microbe-disease predictor	DL	OTU	https://github.com/lichen-lab/mdeep
TaxoNN	Microbe-disease predictor	DL	OTU	https://github.com/divya031090/taxonn_otu.
MetaPheno	Microbe-disease predictor	DL	Microbe data (fasta)	https://github.com/nlapier2/metapheno

## Methods

**Figure 2:**
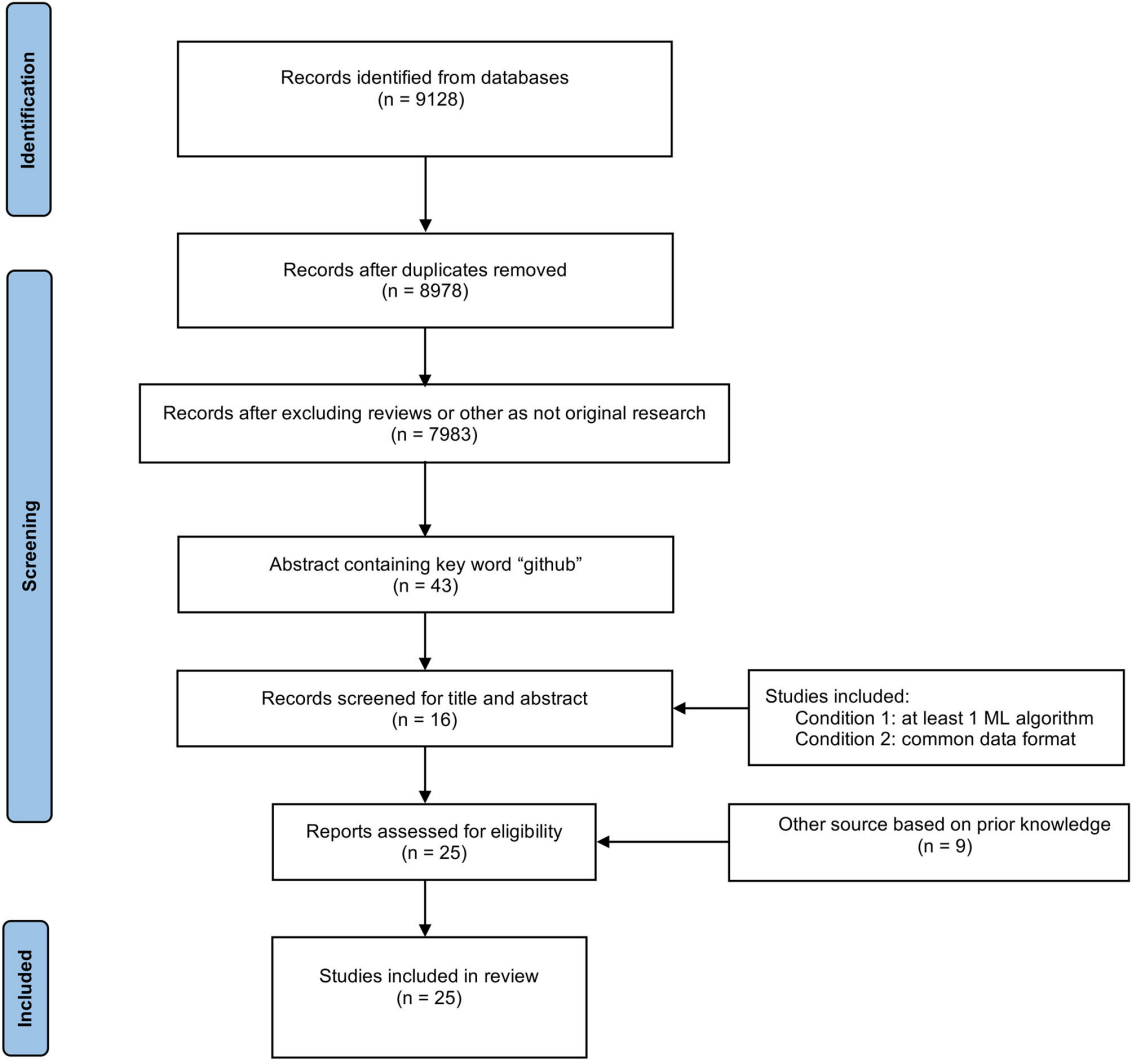
PRISMA flowchart of the review.

We designed a custom semi-automated data collection method incorporating data mining techniques for this mini-review. The PubMed and Google Scholar databases were searched from the inception until March 2022. The search terms used were: “obesity AND machine learning”; “nutrigenetics AND machine learning”; “food recommendation AND machine learning”; “obesity AND geographic information system”; “mhealth” AND (“smartphones” OR “mobile app” OR “mobile applications”) AND (“platform” OR “framework”)”; “(Genetics OR Genomics) AND machine learning”; “microbiome AND machine learning.” The inclusion criteria were as follows: (1) the abstract text contains the keyword “github” (text mining (TM) technology); (2) studies applying at least one ML algorithm focusing on predicting obesity and obesity-related diseases, preventing the obesity prevalence, or conducting the relationship between obesity and obesity risk factors [manually screening (MS)]; (3) the algorithms or frameworks in the studies must be suitable for a common data format, such as fasta or fastq format (MS). Articles excluded were the following: (1) the main text of studies was not available in the English language (TM technology); (2) review papers and papers not considered original studies (TM technology). The PRISMA flowchart of assessment of the literature is illustrated in Figure 2.

## Results

In summary, we included 25 open-source ML algorithms or models applied in obesity in this mini-review. In the following sections, we discuss each algorithm and describe its applications.

### Personalized Food Recommender

A recommendation system can be considered a specified ML model predicting the “rating” or “preference” when given certain information about the user. Deep learning approaches based on the embedding models are now popularly used in modern recommendation systems (15). The core utility of the food recommendation system is to provide recipe retrieval (16). PFoodReq is a novel question-answering food recommendation system based on a large-scale food knowledge base/graph (17). In general, PFoodReq will follow the user's question, such as “What's good with bread for breakfast?”, and then output all recipes from the model. The ingredients in these recipes are then rated for suitability and the top-rated recipes are recommended. FlavorGraph is a knowledge graph (KG) system by relations extracted from food recipes and information on flavor molecules from food databases (18). The two main usages of FlavorGraph are predicting compound food relationships and selecting or optimizing food pairings. Yum-me is a nutrition-based meal recommender that integrates state-of-the-art food image analysis models (19). Its input variables rely on two parts: (I) a survey of user dietary restrictions and nutritional expectations and (II) a visual interface that represents the user's food preferences. DeepFood, based on a deep learning approach, can recognize multi-item (food) images by detecting candidate regions or using a deep convolutional neural network (CNN) for food classification tasks (20). Market2Dish focused on the extension utility of user health profiling and health-aware food recommendation (21).

### Nutrigenetic Model

Nutrigenetics is the science of studying the interaction between nutrition and gene information. LC-N2G is a novel statistical method inheriting genetic algorithm for ranking and identifying combinations of nutrients with gene expression (22). NutriGenomeDB is a nutrigenomics data platform based on the GSEA algorithm, which collects signature gene information of nutrigenomics experimental expression data obtained from the gene expression omnibus (GEO) (23).

### Geographic Information System (GIS) Model

Studying obesity-related issues through GIS data and methods is one of the hotspots in current public health research. A US team pioneered the use of deep learning techniques to assess obesity levels by identifying key patterns in the built environment from satellite imagery (24). The key idea is to extract thousands of hidden features from satellite images and identify the potential relationship between the hidden features and the body mass index (BMI). Unfortunately, the framework is not open to the public. MapMetadataEnrichment, a deep learning–based approach that can automatically generate labeled training map images using GIS data, is highly recommended as an alternative framework (25). Without a deep learning approach, spatial models could not be directly used to analyze GIS data to study obesity (26). In this article, we recommend the GWmodel, which is a widely used R-based package that implements geographically weighted models for exploring spatial heterogeneity (27). A classic use case of the application of the GWmodel can be found in a recent study in which a geographically weighted regression (GWR) model (based on the GWmodel package) was adopted to analyze the relationship between socioeconomic factors, obesity, and air temperature and unhealthy behaviors in the USA (28).

### mHealth Platform

Recently, digital technologies that can monitor and manage our physical and mental health in our daily lives have rapidly developed and are being used to solve obesity-related problems (29, 30). Mobile health applications denoted mHealth apps have become increasingly popular with researchers and clinicians as effective tools for improving health behaviors. Several open-source mHealth platforms are available, including schema (31), mHealthDroid (32), and MobileCoach (33). These platforms are mentioned here since they can cooperate with ML models in the situation where the model uses the data provided by the platform. The mHealhDroid platform is designed to facilitate the fast and easy development of mHealth and biomedical applications; the schema platform is a lightweight cross-platform mobile application focused on mobile health monitoring and intervention research; the MobileCoach platform provides a one-stop solution for fully automated digital interventions.

### Genetic Model

Genetics studies genes and the way certain traits or conditionsare passed from one generation to the next. The polygenic score (PGS) database collects published PGS information that provides the community with an open platform for PGS research. (34). PRS estimates an individual's genetic risk for complex diseases based on many genetic variants across the genome. In fact, a polygenic risk score (PRS) could be considered a specified regression model; there are many obesity PRS models available in the PGS database, such as PGP000017 and PGP000211. Impute.me is the first non-commercial platform for using data from direct-to-consumer genetic testing to calculate and interpret polygenic risk scores (35). DeepVariant was launched by Google, which uses deep neural networks to fast and accurately identify variation sites from DNA sequencing data (36). The NeuralCVD-based deep survival machine algorithm can estimate the cardiovascular risk for coronary heart disease prevention (37). DeepCOMBI uses AI for analysis and discovery in genome-wide association studies (38).

### Microbiome Model

The gut microbiome is closely related to overall health. More and more studies suggest that obesity is associated with specific changes in the composition and function of the human gut microbiome (39, 40). Any study of the gut microbiome associated with obesity cannot skip the step of taxonomic classification to infer the relative abundance of different taxa. Typical ML taxonomic classifiers are DeepMicro (41), DeepMicrobes (42), SortMeRNA (43), q2-feature-classifier (44), swarm (45), etc. We mentioned that q2 feature classifier has been implemented in QIIME 2 which is the most popular microbiome analysis platform (46). ML models [GEDFN (47), MDeep (48), TaxoNN (49), and MetaPheno (50)] can also be used to predict patient phenotype or obesity from their microbiome sequence data. MIPMLP provides a reproducible preprocessing ML pipeline for a microbiome analysis (51).

## Limitations

Obesity, with its complex etiologies, is difficult to describe with a single theoretical model. The current trend in obesity research is to integrate health and medical big data and use high-throughput sequencing technology for multi-omics joint research. In general, the current ML models and algorithms for obesity research are separated from a single field, and no model can span multiple fields or integrate multiple types of data across platforms for a joint analysis of homologous and heterogeneous data. For the nutrigenetic model, the development of open-source models is far behind the industry. Several startups have launched genetics-based food recipes and recommendation systems, while their models are “black box.” A major limitation of genetic models of obesity is weak evidence from small sample sizes due to small variants of genetic mutations. For the mHealth platforms, we could not find open-source ML models in obesity, although we believe that ML models in obesity must be existing in commercial mobile APPs. For the open-source mHealth platform, we were unable to find an open-source ML model related to obesity, although we believe that such ML models already exist in commercial mobile apps.

## Perspective

Machine learning algorithms are a powerful analytic tool that enables us to conceptualize and study metabolic disorders within a fundamentally novel framework. In contrast to conventional statistical methods assessing single modality predictors, ML methods are capable of integrating multiple data types and sources to inform predictive models. Nonetheless, ML algorithms are limited by the type of data captured, the quality of available data, the conceptual frameworks algorithms are applied to, and the underlying assumptions. The use of ML algorithms in obesity will dramatically increase in the future, and a large number of ML algorithms will be implemented on a few specific platforms such as QIMME 2 to facilitate rapid application and collaborative work between multiple algorithms. Presently, most of the ML algorithms used in obesity-related research are isolated in a single field or the factor that causes obesity; in the future, the algorithms will work together across platforms or data types. Future research vistas are to optimize and prospectively test predictive models using external datasets.

## Author Contributions

All authors listed have made a substantial, direct, and intellectual contribution to the work and approved it for publication.

## Funding

This research received financial support from the General Project of the Liaoning Provincial Department of Education under Grant No. LJKZ0758 and the Liaoning Provincial Natural Science Foundation (2021-MS-194).

## Conflict of Interest

The authors declare that the research was conducted in the absence of any commercial or financial relationships that could be construed as a potential conflict of interest.

## Publisher's Note

All claims expressed in this article are solely those of the authors and do not necessarily represent those of their affiliated organizations, or those of the publisher, the editors and the reviewers. Any product that may be evaluated in this article, or claim that may be made by its manufacturer, is not guaranteed or endorsed by the publisher.
